# Preliminary Study Exploring Caretaker Perspectives of Euthanasia on Swine Operations

**DOI:** 10.3390/ani10122296

**Published:** 2020-12-04

**Authors:** Hailey Simpson, Lily N. Edwards-Callaway, Mary Caitlin Cramer, Ivette Noa Roman-Muniz, Lorann Stallones, Sofia Thompson, Sari Ennis, Elizabeth Kim, Monique Pairis-Garcia

**Affiliations:** 1 Department of Animal Sciences, College of Agricultural Sciences, Colorado State University, Fort Collins, CO 80523, USA; hailey.simpson@colostate.edu (H.S.); catie.cramer@colostate.edu (M.C.C.); noa.roman-muniz@colostate.edu (I.N.R.-M.); sofia464464@gmail.com (S.T.); 2 Department of Psychology, Colorado State University, Fort Collins, CO 80523, USA; lorann.stallones@colostate.edu (L.S.); sari.ennis@colostate.edu (S.E.); elizabeth.kim@colostate.edu (E.K.); 3 Department of Population Health and Pathobiology, College of Veterinary Medicine, North Carolina State University, Raleigh, NC 27607, USA; pairis-garcia@ncsu.edu

**Keywords:** caretaker, euthanasia, pig, training, worker well-being

## Abstract

**Simple Summary:**

Euthanasia is utilized on livestock operations as a mechanism to alleviate suffering of diseased or injured animals that have little chance of recovery. Although euthanasia decisions are made with the animal’s best interests in mind, it is often a difficult job for caretakers to perform. Research exploring the impacts of performing euthanasia on people who have chosen careers based on their affinity for caring for animals has been more prevalent in veterinarians and animal shelter workers as compared to livestock caretakers. The intent of this study was to gain a preliminary understanding of caretaker perspectives of euthanasia-related training, support, and resources available on swine operations. Although most caretakers felt confident performing euthanasia, some identified the desire to have more euthanasia training. Human safety was consistently included in euthanasia training, as it should be, but strategies to cope with personal stress and ensure emotional wellness related to this specific task were not as reliably incorporated. Although the majority of caretakers indicated that there were programs to promote worker health and resources to help them cope with job responsibilities, there is still an opportunity to integrate components of mental wellness into training and on-farm support programs.

**Abstract:**

The purpose of this study was to gain a better understanding of euthanasia training, caretaker perceptions of euthanasia, and available resources for individuals who perform euthanasia as part of their job on swine operations in the United States. An online survey was distributed via an e-newsletter and in-person recruitment at swine industry events. Survey questions were related to training, attitudes towards performing euthanasia, work environment, and communication. Forty-five responses (17 workers, 21 managers, 7 owners) were recorded and summarized. The majority of workers (*n* = 14, 82%) agreed that they had “received enough training to euthanize pigs correctly” and that training had made them “confident about performing euthanasia”, yet 35% (6) also indicated they would like more euthanasia training. Less than one-third of workers indicated that strategies for dealing with “personal stress” (4, 24%) and “emotional wellness” 29% (5) were included in training programs but the majority (14, 82%) agreed that “trainings included human safety while performing euthanasia”. Most caretakers (37, 82%) agreed that they felt they could “communicate with my supervisors” if they felt uncomfortable performing euthanasia. Opportunities for the future include enhancing euthanasia training opportunities and content to include more awareness of strategies to deal with stress related to euthanasia.

## 1. Introduction

Euthanasia is a management tool utilized on livestock operations to alleviate animal suffering. It is critical that euthanasia decisions are made in a timely manner and performed by trained individuals in a way that minimizes pain and distress for the animal. Guidelines for approved euthanasia methods are published by national organizations such as the American Veterinary Medical Association (AVMA) [1] in addition to species-specific associations; in swine, the National Pork Board (NPB) and the American Association of Swine Veterinarians (AASV) develop and provide these materials for producers [2]. Animal caretakers often participate in both making euthanasia decisions and performing euthanasia [3,4,5,6,7,8] and therefore having clear protocols, procedures, and training in place is essential. The NPB Pork Quality Assurance (PQA) Plus^®^ program emphasizes both the necessity of caretaker euthanasia training and written and accessible standard operating procedures (SOPs) on farm [9]. 

There are a multitude of factors impacting euthanasia decisions on livestock operations, including but not limited to inconsistent caretaker training, lack of written SOPs, treatment decisions, and the human–animal bond [10]. The emotional impact of euthanasia on animal caretakers can be significant [11,12,13,14,15,16,17]. Although “compassion fatigue” has been a focus of research in companion and shelter veterinary medicine, there is limited exploration about this type of stress in livestock caretakers. Compassion fatigue is often referred to as the “cost of caring” and explains the physical and psychological strain of being repeatedly exposed to another’s pain and suffering, both relevant to those who care for other humans and/or animals [18,19]. Rollin [20] describes another concept, “the caring-killing paradox,” which explains the conflict of caretakers who enter their line of work to benefit animals and minimize harm to them, yet are faced with making euthanasia decisions which, even when alleviating suffering, oppose this goal. It has been shown that animal shelter workers and veterinarians that are asked to euthanize animals regularly experience adverse feelings including guilt, regret, negative feelings towards themselves and symptoms related to traumatic stress [12,13,14,21]. 

In previous studies in both companion and livestock species, animal caretakers have identified that they understand the importance of euthanasia despite the emotional stress it may cause [6,8,13,15,17,20,22]. There is an opportunity in the swine industry to explore ways to provide animal caretakers with strategies to deal with the emotional stress of having to euthanize animals that they care for. Rogelberg et al. [23] reported that for animal shelter workers, provisions of counseling, time off, and communication were needed for those performing euthanasia regularly to prevent fatigue and improve employee health. In livestock workers, it has been shown that guidance and training about euthanasia can greatly impact the feelings an animal caretaker has about their work [17,24]. High quality support networks or resources available to caretakers impacts their ability to cope with job-related stress [14]. The impact these resources can have on the well-being of farmers directly impacts the animal welfare treatment on farms as well as job performance [25,26]. Improving the well-being of swine caretakers who perform euthanasia would have a positive effect on both worker and animal well-being, which would also positively impact operation efficiency and sustainability.

Understanding caretaker perceptions about euthanasia and associated training, communication, and available resources will provide valuable information that can guide future euthanasia resource and program development both on farm and broadly on an industry level. The purpose of this study was to gain knowledge on euthanasia training, caretaker perceptions of euthanasia, and available resources for caretakers who perform euthanasia as part of their job on swine operations. 

## 2. Materials and Methods 

This research was approved through the Colorado State University (CSU) Institutional Review Board (#19-9050H) prior to project initiation. The data reported in this paper are from a larger survey that also included swine veterinarians and the methodology is similar to that reported in Edwards-Callaway et al. [6]. 

### 2.1. Study Population and Recruitment

The target population for this survey was caretakers who work on swine operations and are involved in euthanasia management (e.g., decision making, training, performance). In this study, caretakers on swine farms were defined as owners, managers and workers. Study recruitment occurred either via online outlets (email and newsletters) or in-person events beginning in November 2019 and extending to February 2020. The survey was offered as a phone interview initially but due to minimal uptake, the survey was converted to an online platform. Survey information and recruitment materials were shared with swine production companies directly. Additionally, the survey was announced in the weekly AASV e-newsletter (1585 subscribers) two times in an effort to share the survey information broadly. Three co-authors attended the Pig Welfare Symposium in Minneapolis, MN in November 2019 (312 attendees) and hosted a booth at which recruitment flyers were distributed to attendees with the survey information. In February 2020, a co-author attended the Illinois Pork Expo in Springfield, IL (837 attendees) to recruit using the same method as the former event. 

Respondents were offered a $25 gift card for participation. Respondents received compensation by electing to leave an email address at the end of the survey. All responses remained anonymous and no identifying information was associated with responses given. Respondents were eligible to opt out of questions that they did not want to answer. The survey was available in either Spanish or English and respondents were asked to select their preferred language at the start of the survey.

### 2.2. Survey Questions and Format

All study collaborators were well-versed in survey question development and qualitative data analysis and had expertise in animal science, veterinary medicine, public health and epidemiology. The survey was developed in Qualtrics software (Qualtrics, Provo, UT, USA). Prior to study initiation, the survey was reviewed by all co-authors in addition to external individuals involved in some sector of the livestock industry (e.g., veterinarians, professors, producers) to check survey functionality and clarity. The survey used a branching method based on job title or role (manager, worker, owner, or veterinarian; veterinarian information reported in Edwards-Callaway et al. [6]) and therefore depending upon answers, respondents were asked different total numbers of questions. The survey was intended to take less than 30 min to complete. The entire worker survey is provided as Supplementary Materials (File S1). A variety of question types were used including dichotomous, multiple-choice, Likert scale, rating scale, and open-ended questions. There were six categories of questions within the survey: euthanasia method, frequency, and training; job satisfaction and well-being; attitudes towards performing on-farm euthanasia; management attitude; support networks; and demographic and background information. 

Three phone interviews were conducted. A co-author conducted all of these interviews; all survey questions were asked to the respondents and answers were recorded. The questions asked during the phone interview were identical to those included in the online survey.

### 2.3. Statistical Analysis

After the survey was closed, data were exported to a Microsoft Excel (Microsoft Corporation, Redmond, WA, USA) spreadsheet and reviewed by two researchers for data entry errors and completeness. Fifty-nine surveys were received and surveys less than 80% complete were not used for analysis (*n* = 14). Forty-five surveys were included in final results. Some respondents either declined to answer or provided no answer for various questions; these categories were noted in all data summaries where applicable. Descriptive statistics were tabulated for all questions of interest. For agreement statements, respondents who selected “agree” or “strongly agree” were represented as “agree” and this was similarly done for “disagree” and “strongly disagree.” Due to the relatively small sample size, no additional statistical analysis was completed.

## 3. Results

A total of 59 surveys were returned. Considering the number of attendees at the Pig Welfare Symposium (312 attendees) and Illinois Pork Expo (837 attendees), it is estimated that the survey had a response rate of 5% which is likely an overestimation due to other outlets through which the survey was shared that are not easily quantified (e.g., direct emails to producers). An additional consideration is that many of the conference attendees may not have fit the criteria for survey participation and therefore this response rate is merely an estimate. Considering the low response rate, these data should be considered preliminary and should be followed by studies that capture a larger proportion of the target population.

### 3.1. Demographics and Background Information

Respondent demographics are shown in Table 1. Swine caretakers consisted of 17 workers, 21 managers and 7 owners, all of whom identified that they worked with pigs occasionally or often. The sample was 58% (*n* = 26) male and 42% (19) female reporting an average age of 37 years. Most of the respondents were from the Midwest (32, 71%); U.S. regions as defined in O’Connor [27]. The majority of respondents (30, 67%,) indicated that their native language was English and were non-Hispanic or non-Latino (30, 67% and 32, 71%, respectively). Approximately half (25, 56%) of respondents said they had not previously worked on a pig farm before their current job. Respondents were asked to report their highest level of education to which a Bachelor’s degree was the most frequently selected answer (19, 42%) with the rest of the respondents varying in reported education levels.

### 3.2. Euthanasia Method and Training

A branching methodology was utilized in this study, and therefore, workers, owners and managers were asked different questions. These questions varied slightly in context to correspond to the specific role of the respondent on the farm where they worked. In doing so, most questions relating to the methods of euthanasia and training were only available for respondents that identified as workers (*n* = 17). 

The most highly reported method of euthanasia used for sows was penetrating captive bolt (8, 25%), but gunshot (5, 16%) and electrocution (1, 3%) were also selected as methods used. Non-penetrating captive bolt was the most highly reported method for euthanasia identified for piglets (8, 26%). Carbon dioxide (4, 13%), penetrating captive bolt (2, 7%), blunt force trauma (3, 10%) and electrocution (1, 3%) were also selected as other methods used for piglet euthanasia. In sows, no respondents indicated that they felt discomfort associated with any euthanasia method when asked “If you euthanize sows, does performing euthanasia using this method cause you discomfort?”. When asked the same question for piglets, at least one respondent indicated feeling discomfort to all methods except carbon dioxide.

Workers (*n* = 17) were asked to provide information about the frequency of training and the type of training used for any method of euthanasia utilized on their farm (this question was asked to all respondents but none of the manager or owner surveys that were considered complete answered this question). On average, respondents reported that training occurred one time per year for all methods of euthanasia used for both sows and piglets. Respondents identified that training was offered in English, Spanish, and sometimes both. 

Questions about perceptions of training were asked in the form of agreement statements (Table 2). The majority of workers agreed with the statements “training has made me confident about performing euthanasia” and “I have received enough training to euthanize pigs correctly” (14, 82%, for both). Additionally, the majority of workers agreed that “the frequency of training is adequate” and that “training is delivered in a format that helps me learn” (15, 88% and 16, 94%, respectively). The majority of respondents also (14, 82%) agreed that “trainings included human safety while performing euthanasia”. Almost one-quarter (4, 24%) of respondents agreed that their trainings included “strategies to cope with personal stress” and only 29% (5) agreed that their trainings included “strategies for emotional wellness”. Nearly one-third (6, 35%) of respondents said they “would like to receive more euthanasia training”. 

### 3.3. Euthanasia Decision Making

All respondents (*n* = 45) were asked to identify who on their farm makes the decisions to euthanize and additionally who performs euthanasia (Table 3). Respondents were able to select multiple answers to these two questions. Approximately half of respondents indicated that they themselves (22, 49%) made the decision to euthanize. Approximately forty percent of respondents indicated that a caretaker other than themselves (18, 40%), and/or a manager (16, 36%) also participated in euthanasia decision making. When asked whether they “perform euthanasia by yourself of with another staff/team member” most respondents selected “by yourself” (26, 58%) and/or “with staff member” (21, 47%).

### 3.4. Attitudes Towards Performing On-Farm Euthanasia

**Figure 1:**
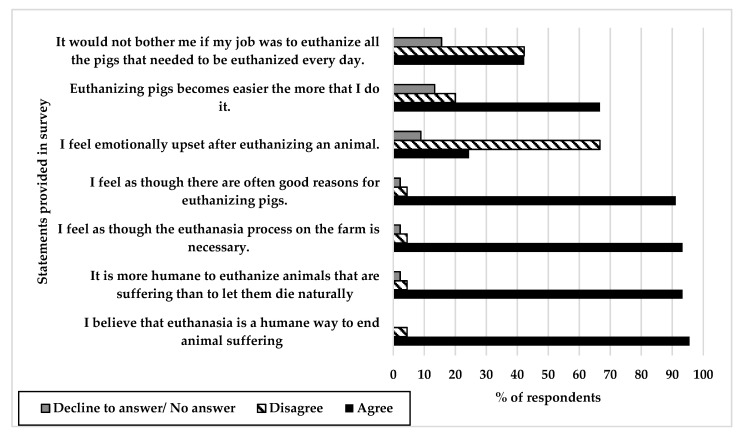
The level of agreement by respondents (*n* = 45) with various statements regarding perceptions of euthanasia. For each statement, respondents were given the following options: strongly agree, agree, disagree, strongly disagree, and decline to answer. In this figure, “Agree” represents agree and strongly agree responses and “Disagree” represents disagree and strongly disagree responses. “Decline to answer” also includes respondents that provided no selection.

Figure 1 shows the level of agreement with statements regarding caretakers’ perceptions of euthanasia (*n* = 45). Most respondents disagreed with the statement “I feel emotionally upset when performing euthanasia” (30, 67%) but almost half (19, 42%) indicated that “it would bother me if my job was to euthanize all the pigs that needed to be euthanized every day” and the majority (30, 67%) agreed that “euthanizing pigs becomes easier the more that I do it”. Ninety-six percent (43) of respondents agreed that “euthanasia is a humane way to end an animal’s suffering” and 93% (42) said that “it was more humane to euthanize an animal that is suffering rather than let them die naturally.” Nintey-one percent (41) of caretakers agreed that “there are often good reasons for euthanizing pigs” and 93% (42) agreed with the statement “I feel as though the euthanasia process on the farm is necessary”.

### 3.5. Work Environment and Communication

Table 4 includes information regarding mental health resources, communication and work environment (*n* = 45). The majority of respondents (24, 53%) indicated that there are “programs to promote worker health” (Table 4). Similarly, 58% (26) of respondents indicated that there were “employee check-ins with a supervisor or administrator”. Thirty-three respondents (73%) indicated that there were not any “mental health evaluations” at their place of work.

**Figure 2:**
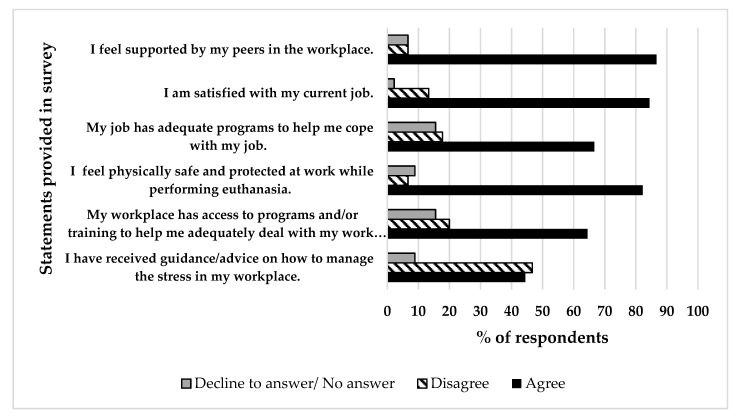
The level of agreement by respondents (*n* = 45) with various statements regarding perceptions of the workplace. For each statement, respondents were given the following options: strongly agree, agree, disagree, strongly disagree, and decline to answer. In this figure, “Agree” represents agree and strongly agree responses and “Disagree” represents disagree and strongly disagree responses. “Decline to answer” also includes respondents that provided no selection.

Figure 2 shows the responses to questions related to participant perspectives about the workplace. Approximately three-quarters of the respondents agreed that their workplaces had “access to programs and/or training to help me deal with my work responsibilities” and had “adequate programs to help me cope with my job” (29, 64% and 30, 67%, respectively). Nearly half of the respondents (20, 44%) said they had received “guidance or advice on how to manage stress in my workplace”. A majority of respondents said they felt “physically safe at work while performing euthanasia” (38, 84%), that they were “satisfied with my current job” (38, 84%) and that they felt “supported by my peers in the workplace” (87%, 39).

A majority (37, 82%) agreed that they felt they could “communicate with my supervisors if I feel uncomfortable performing euthanasia” and 89% (40) said they were “aware of proper channels in management to communicate issues” (Table 5). The majority of caretakers (42, 93%) said they agreed that their “supervisors aimed to promote a safe and encouraging work environment”. 

When asked whether respondents talked to people about work and who they talked to about feelings on performing euthanasia, respondents indicated that they most commonly talked to work peers (7 work peers on average). 

## 4. Discussion

The results summarized in this study represent forty-five swine caretakers, a relatively small sample size. Reaching individual animal caretakers that met the criteria for the survey was a challenge and future studies should explore innovative recruitment techniques (e.g., social media posts), different survey delivery mechanisms (e.g., in-person on paper vs online) or shorter survey lengths. To be successful in future studies that look to engage with livestock workers, it is suggested that conversations occur between relevant industry stakeholders to both discuss the importance of the work and to brainstorm new approaches to engaging this critical group of caretakers. Additionally, we would like to acknowledge that results combine all respondent responses, which includes managers, owners and workers. It would be beneficial for future work, ideally with larger sample sizes, to explore the differences in perspectives between these different categories of caretakers.

The majority of the respondents were non-Hispanic and spoke English as their native language. The 2015–2016 US Department of Labor National Agricultural Workers Survey (NAWS) reported that within the agricultural industry, over three-quarters of agricultural workers primarily speak Spanish [28]. The NAWS report also indicated that, nationally, agricultural workers are predominantly male (68%) with an average age of 38 [28]; the current study population aligns with reported national age and gender statistics for agricultural workers. Nationally, 35% of agricultural workers have completed some adult education [28] as compared with the current study population in which 42% had attended some college and 22% had a post-graduate degree (other than veterinary school). These differences in study population demographics as compared with national statistics of agricultural workers highlights the potential bias in participant recruitment methods. Attendees at the two industry events, where a substantial amount of recruitment occurred, likely did not represent the US population of swine caretakers but rather this subpopulation probably came from progressive operations, held managerial or supervisory roles, and attended more years of formal education. These limitations should be considered when evaluating study conclusions. 

One of the objectives of this project was to gain information about euthanasia training on pig farms. The PQA Plus^®^ handbook specifies that euthanasia training should be documented and that caretakers should be able to explain: the method of euthanasia, the handling of animals during the euthanasia process, and insensibility and confirmation of death [9]. The PQA Plus^®^ handbook also indicates that a written SOP for euthanasia should be accessible on farm; although not stated in the handbook, accessibility means both visually available and provided in the native language of individuals performing the particular task [4]. 

The majority of workers participating in this study agreed that “all employees performing euthanasia have been trained adequately” and that they themselves have“received enough training to euthanize pigs correctly” Follow-up questions were not asked to determine why respondents felt training was adequate but other agreement statements indicated that worker respondents agreed that “the frequency of training is adequate” and “training is delivered in a format that helps me learn” pointing to frequency and delivery format as relevant training components for learners. Previous research, specifically with swine caretakers, has indicated that on-farm training is preferred over other platforms (e.g., written materials or videos), but that supplemental classroom instruction is still valuable in education on euthanasia [29]. Additionally, workers in this study indicated that they feel euthanasia becomes easier the more it is performed; this relates to comfort with the task but also the skill that comes with continued hands-on experience. Similar results related to increased comfort with increased experience has been reported in veterinarians [6]. Recent research efforts have explored the use of innovative delivery methods for euthanasia training with swine caretakers (i.e., interactive computer-based training) with success in improving knowledge post-training [8]. In regards to frequency, respondents in the current study indicated that on average euthanasia training occurred once a year. Many industry guidelines and assessment tools require annual training for livestock caretakers, with euthanasia training being a component of general animal care training [9,30,31]. The optimal frequency of training needed to optimize knowledge retention, skill, and confidence specific to making decisions about and performing euthanasia has not been extensively explored. In a review of factors impacting training in Spanish-speaking livestock workers, Roman-Muniz et al. [32] outlined the importance of many factors essential to maximizing the benefit of training initiatives, including consistent follow-up and retraining. Langley and Morrow [33] identified the importance of conducting training multiple times in part due to staff turnover and subsequent on-boarding of new employees. There is opportunity to further explore factors that impact the effectiveness of training and retraining on specific outcomes related to both job performance and worker attitudes.

Workers were asked to select all methods of euthanasia training they had received by selecting from a provided list of training options. Respondents indicated the use of multiple training methods including in-person outside trainer, in-person in-house trainer, videos, online materials, written materials, and shadowing a co-worker. As animal caretakers become more familiar with the euthanasia process via training and review of protocols, the stress of performing the task decreases [3]. Turner and Doonan [3] suggest that the euthanasia protocol should be readily available for caretakers and that training should include explanation of the dying process so that individuals are prepared for what they will observe. Matthis [34] indicated that an understanding of the physiological components of euthanasia could improve comfort level for workers who deal with euthanasia and thus improve job satisfaction. For training to be effective in adult learners, regardless of the topic area, they must understand ‘why’ things need to be done a certain way to appreciate the value and importance of their work [24]. This comprehensive approach to including both the ‘how’ and the ‘why’ should be considered in future euthanasia training development efforts within the livestock industry.

Respondents reported that English was the primary language used for training across all methods of euthanasia for both sows and piglets. This would be expected as over half (67%) of respondents said their primary language was English. For the other one-third of respondents that defined themselves as non-native English speakers, there were limited data as to what language they were trained in (due to unanswered questions). One-third of the non-native English speakers indicated that training was offered in both English and Spanish. Most non-native English speakers (77%, 10) identified their proficiency in English as “I speak it well” when asked about their second language proficiency. As stated, almost three-quarters of agriculture workers are primarily Spanish speaking [28] so it would be important to offer trainings and communication in Spanish as needed on operations. The US swine industry has animal care education resources available in Spanish [35,36] but some other livestock associations that develop and produce animal care guidelines and training resources only have English versions or very limited Spanish versions available [37,38], with current efforts underway to have Spanish materials made available. The importance of culturally congruent training on dairy operations is emphasized in previous research and among other essential components the importance of offering bilingual training when necessary has been emphasized [32,39,40,41]. Priority should be placed on updating training materials to address the needs of the workers. 

Despite the fact that most workers indicated that euthanasia training was adequate, approximately one-third of respondents indicated that they would like to have more euthanasia training. Similarly, in other studies in swine and dairy caretakers, individuals have indicated a desire for more training with particular mention of in-person, on-the-job training [29,34,39,42]. In similar survey studies with livestock veterinarians, the participating veterinarians have indicated the desire to conduct more euthanasia training [4,6]. Furthermore, the importance of including livestock veterinarians in on-farm euthanasia training and protocol development has been articulated in multiple studies [3,10,32]. This shared desire to participate in more training both from the perspective of the trainer and the trainee identifies an area of opportunity to enhance current euthanasia training programs and strengthen veterinarian involvement. 

Worker respondents were asked whether they agreed with statements about the inclusion of “human safety,” “strategies to cope with personal stress”, and “emotional wellness” in euthanasia training. All worker respondents except two (one disagreed and one did not provide an answer) indicated that human safety is included in euthanasia training. General health (physical) and safety of livestock workers on farm, primarily related to on-the-job injury, respiratory conditions, and zoonotic disease exposure [33,39,41,43,44,45,46,47] have received relatively more attention in regards to preventative measures and program development as compared to some other aspects of worker well-being, i.e., mental health. Less than one-third of survey respondents indicated that strategies for dealing with “personal stress” and “emotional wellness” are included in training programs. Similarly, in a related study with swine veterinarians, just over half of the respondents indicated that these components were not included in euthanasia training [6]. As mentioned, the moral stress that comes from having to perform euthanasia has been continually acknowledged so the inclusion of techniques for addressing this stress in euthanasia training is an area in need of attention.

The vast majority of respondents identified in their responses to agreement statements about euthanasia that they felt there were “good reasons for euthanizing pigs”, that the process “is necessary”, and that it was a “humane way to end animal suffering”. These sentiments are often identified by caretakers as helpful in dealing with the stress of euthanasia, i.e., acknowledging the importance of euthanasia for animals in need [13,15,20,22]. It is notable that most respondents also articulated that euthanasia is a more humane option as compared to natural death; this is an important finding as it may be a helpful discussion point when teaching caretakers about making timely euthanasia decisions on farm. Despite the difficulty in performing euthanasia, individuals still respect and understand the necessity of having this option available to end animal suffering. Interestingly, the majority of respondents shared that they did not “feel emotionally upset after euthanizing animals” and just under half also indicated that they would not be bothered whether their job was to “euthanize all the pigs that needed to be euthanized every day”. Further questioning was not included to determine why respondents felt this way but it is thought that these feelings relate to a caretaker’s sense of duty and empathy towards the animals under their care.

Over half of the respondents indicated that the farms they worked on had programs to promote worker health and that there were employee check-ins with supervisors. Although this is the majority, almost one-third of respondents indicated that these resources were not available, which is concerning. Even fewer respondents (16%) identified that mental health evaluations were a part of workplace programming. As mentioned, physical health and safety is often addressed at the workplace; in this study, every respondent agreed that their “supervisors aim to promote a safe an encouraging work environment.” It is critical to consider inclusion of other aspects of worker wellness into training and health and safety programming. Particularly in the context of this paper, euthanasia can be a source of emotional stress for animal caretakers and therefore more attention should be given to resource development relevant to this specific type of job-related stress. 

When asked more detailed questions about communication, the majority of respondents indicated that they understand who to communicate with and feel comfortable communicating with supervisors whether they feel uncomfortable performing euthanasia, suggesting a positive work environment. Additionally, in responses to agreement statements about perceptions of the workplace, the majority of respondents once again provided feedback that they feel “supported by peers”, “physically safe and protected at work”, feel “satisfied with my current job” and “have access to programs and/or training to help me adequately deal with my work responsibilities.” It is important to note that although the majority of respondents shared positive feelings about the workplace, there were a few individuals that did not and it is important to consider perceptions of all employees. Additionally, when asked about “receiving guidance/advice on how to manage stress at the workplace”, almost half of the respondents indicated they did not agree this type of guidance was provided. This feedback supports previous discussion about the importance of developing and providing resources to caretakers to support them in dealing with potentially stressful parts of their job, e.g., euthanasia. The availability of resources, support, and stress management tools will improve worker well-being, which will concurrently positively impact an organization’s effectiveness through enhanced job satisfaction [48,49,50]. 

## 5. Conclusions

Although these results are from a limited sample size, this work offers valuable insight into swine caretaker perspectives that can be used in future research and evaluation of euthanasia practices on farms. The results indicate that although the euthanasia training on the worker respondents’ respective farms was thought to be adequate, there is a desire for further training on this topic. Additionally, based on indications of what was included in euthanasia training, the incorporation of strategies to cope with the mental stress of performing euthanasia should be considered for on-farm euthanasia training. While this survey was specific to swine caretakers, the focus on the potential emotional impacts of euthanasia on caretakers should be given more attention across livestock operations. Resources for physical and mental health should be readily available to workers to promote worker well-being, improved job performance, and greater job satisfaction. It appears that there is already a foundation for health programming in swine operations but there should be an emphasis on both the physical and mental aspects of employee health; this is especially relevant in the context of euthanasia. Lastly, it is important to acknowledge that caretakers understand and appreciate the importance of euthanasia on farm as a way to reduce the suffering of certain animals and thus it is critical that they are provided with the appropriate resources needed to help them cope with this part of their job.

## Figures and Tables

**Table 1:** Demographics of survey respondents (*n* = 45).

Role	Respondents % (*n*)
Worker	38 (17)
Manager	47 (21)
Owner	16 (7)
Gender	
Male	58 (26)
Female	42 (19)
Average Age (years)	
	37
Location of Current Residence ^1^	
Midwest	71 (32)
Southwest	7 (3)
West	7 (3)
Southeast	9 (4)
Outside of North America	4 (2)
No answer	2 (1)
Ethnicity	
Hispanic or Latino	24 (11)
Non-Hispanic or Non-Latino	71 (32)
Decline to answer	4 (2)
Native Language	
English	67 (30)
Spanish	24 (11)
Portuguese	2 (1)
Ukrainian	2 (1)
No response	4 (2)
Previous Work on a Pig Farm	
Yes	40 (18)
No	56 (25)
Decline to answer	4 (2)
Highest Level of Education	
No high school diploma	2.2 (1)
High school diploma	13 (6)
Some college	13 (6)
Bachelor’s degree	42 (19)
Veterinary degree	7 (3)
Post-graduate degree (other than veterinary school)	22 (10)

^1^ Regions were defined as quoted by O’Connor [27]. Both languages. The language the training was offered in varied by euthanasia method. Respondents indicated receiving training in the following formats: in-person outside trainer, in-person in-house trainer, video, online, written materials, and shadowing a co-worker.

**Table 2:** The level of agreement by respondents identified as workers (*n* = 17) with various statements regarding perceptions of euthanasia training. For each statement, respondents were given the following options: strongly agree, agree, disagree, strongly disagree, and decline to answer. In this table, “Agree” represents agree and strongly agree responses and “Disagree” represents disagree and strongly disagree responses.

Survey Agreement Statements	Respondents % (*n*)		
	Agree	Disagree	Decline to Answer/No Response
I have received enough training to euthanize pigs correctly.	82 (14)	12 (2)	6 (1)
The frequency of training is adequate.	88 (15)	6 (1)	6 (1)
Training is delivered in a format that helps me learn.	94 (16)	0	6 (1)
All employees performing euthanasia have been trained adequately.	88 (15)	6 (1)	6 (1)
Training has made me confident about performing euthanasia.	82 (14)	6 (1)	12 (2)
Training includes human safety while performing euthanasia.	82 (14)	6 (1)	12 (2)
Training includes strategies to cope with personal stress.	24 (4)	71 (12)	6 (1)
Training includes strategies for emotional wellness.	29 (5)	65 (11)	6 (1)

**Table 3:** The roles of people responsible for making the decision to euthanize animals and perform it as identified by survey respondents (*n* = 45). Respondents were able to choose more than one answer for each question.

Who Makes the Decision to Euthanize? (Multiple Select Answer)	% (*n*)
Respondent	49 (22)
Caretaker (other than yourself)	40 (18)
Manager (other than yourself)	36 (16)
Veterinarian	4 (2)
Owner (other than yourself)	9 (4)
Other	9 (4)
Not applicable	2 (1)
**Do You Perform Euthanasia by Yourself or with Another Staff Member/Team? (Multiple Select Answer)**	
By yourself	58 (26)
With staff member	47 (21)
No response	6 (3)

**Table 4:** Questions related to availability of health resources (*n* = 45).

Survey Questions	Respondents % (*n*)		
	Yes	No	Decline to Answer/No Response
Are there programs to promote worker health?	53 (24)	38 (17)	9 (4)
Are there any mental health evaluations?	16 (7)	73 (33)	11 (5)
Are there employee check-ins with a supervisor or administrator?	58 (26)	33 (15)	9 (4)

**Table 5:** Agreement statements related to work environment and communication in the workplace (*n* = 45).

Survey Agreement Statements	Respondents % (*n*)		
	Agree	Disagree	Decline to Answer/No Response
I feel as though I can communicate with my supervisors if I feel uncomfortable performing euthanasia.	82 (37)	9 (4)	9 (4)
I am aware of proper channels to communicate issues to management.	89 (40)	2 (1)	8 (4)
My supervisors aim to promote a safe and encouraging work environment.	93 (42)	0	7 (3)

## Supplementary Materials

The following are available online at https://www.mdpi.com/2076-2615/10/12/2296/s1, File S1: Survey Questions.
