# Supplementary material for: Preliminary Study Exploring Caretaker Perspectives of Euthanasia on Swine Operations
Source: Animals (Basel). 2020 Dec 4;10(12):2296. doi: 10.3390/ani10122296 (PMC7761904; doi:10.3390/ani10122296)
Supplement: Supplementary file 1 [file animals-10-02296-s001.pdf]

NPB Master Survey

A Please type your first name:

____

B Please type the complete name of the state in which you reside :

____

C How would you describe your current role in the swine industry?

- ○ Owner – works with pigs occasionally
- ○ Owner – works with pigs often
- ○ Manager – works with pigs occasionally
- ○ Manager – works with pigs often
- ○ Caretaker – works with pigs occasionally
- ○ Caretaker – works with pigs often
- ○ Veterinarian – works with pigs occasionally
- ○ Veterinarian – works with pigs often
- ○ Declined to answer question

Q1 Is performing euthanasia one of your routine job tasks?

- ○ Yes
- ○ No
- ○ Not applicable
- ○ Decline to answer

Q2 Approximately how many **sows** do you work with daily? Please specify a number or N/A:

____

Q3 How often do you euthanize a **sow**?

- ☐ How many daily ____
- ☐ If not daily, how many weekly ____
- ☐ If not weekly, how many monthly _____________________________________
- ☐ If not monthly, how many annually _____________________________________
- ☐ Not applicable
- ☐ Decline to answer

Q4
Approximately how many **piglets** do you work with daily? Please specify a number or N/A:

____

Q5 How often do you euthanize a **piglet**?

- ☐ How many daily ____
- ☐ If not daily, how many weekly _______________________________________
- ☐ If not weekly, how many monthly ______________________________________
- ☐ If not monthly, how many annually ____________________________________
- ☐ Not applicable
- ☐ Decline to answer

Q6 Do you perform euthanasia by yourself or with another staff member/team? If both, select both boxes. If with a staff member, identify position.

- ☐ By yourself
- ☐ With staff member ____
- ☐ Not applicable
- ☐ Decline to answer

Q7 Who makes the decision to euthanize?

- ▢ Owner (other than yourself)
- ▢ Manager (other than yourself)
- ▢ Caretaker (other than yourself)
- ▢ You
- ▢ Veterinarian
- ▢ Other ____
- ▢ Not applicable
- ▢ Decline to answer

Q8 What determines which euthanasia method is used?

____

____

____

____

Q9 If during your day, you identify an animal that needs to be euthanized, explain the process. What are some unexpected challenges that you did not anticipate when taking this job?

____

____

____

____

____

Q10
If you euthanize <u>sows</u>, please answer the following questions:
Questions related to type of euthanasia and frequency of use:

	Do you perform this method of	Does performing euthanasia using this method	Frequency of Use	Does performing euthanasia

	euthanasia in your current role			cause you discomfort?								using this method cause you discomfort?
	Yes	No	Decline to answer	Yes	No	Decline to answer	How many daily	If not daily, how many weekly	If not weekly, how many monthly	If not monthly, how many annually	Decline to answer	If yes, please explain why:
Gunshot												
Penetrating captive bolt												
Electrocution												
Anesthetic overdose												

Q11

If you euthanize <u>sows</u>, please answer the following questions:

Questions related to training associated with various methods of euthanasia:

	Have you been trained to perform this method?			Frequency of Training		What language was the training offered in?					What language was the training offered in?	Type of training (select all that apply)								Type of training
	Yes	No	Decline to answer	How many times per year do you receive training for this type of euthanasia?	Decline to answer	Spanish	English	Both	Other	Decline to answer	If Other was selected, specify	in-person outside trainer	in-person in-house trainer	video	online	written materials	shadowing a co-worker	Other	Decline to answer	If Other was selected, specify
Gunshot																				
Penetrating captive bolt																				
Electrocution																				
Anesthetic overdose																				

Q12

If you euthanize <u>piglets</u>, please answer the following questions:

Questions related to type of euthanasia and frequency of use:

	Do you perform this method of euthanasia in your current role?			Does performing euthanasia using this method cause you discomfort?			Frequency of Use					Does performing euthanasia using this method cause you discomfort?
	Yes	No	Decline to answer	Yes	No	Decline to answer	How many daily	If not daily, how many weekly	If not weekly, how many monthly	If not monthly, how many annually	Decline to answer	If yes, please explain why:

Carbon dioxide												
Non-penetrating captive bolt												
Penetrating captive bolt (>12lbs)												
Electrocution (> 3 days)												
Anesthetic overdose												
Blunt force trauma (												

Q13

If you euthanize piglets, please answer the following questions:
Questions related to training associated with various methods of euthanasia:

	Have you been trained to perform this method?			Frequency of Training		What language was the training offered in?					What language was the training offered in?	Type of training (select all that apply)								Type of training
	Yes	No	Decline to answer	How many times per year do you receive training for this type of euthanasia?	Decline to answer	Spanish	English	Both	Other	Decline to answer	If Other was selected, specify	in-person outside trainer	in-person in-house trainer	video	online	written materials	shadowing a co-worker	Other	Decline to answer	If Other was selected, specify
Carbon dioxide																				
Non-penetrating captive bolt																				
Penetrating captive bolt (>12lbs)																				
Electrocution (> 3 days)																				
Anesthetic overdose																				
Blunt force trauma (																				

Q14
The following questions relate to training. Please rate your experience with each topic by

checking the appropriate box.

	Strongly Disagree	Disagree	Agree	Strongly agree	Decline to answer	Not applicable
I have received enough training to euthanize pigs correctly.	○	○	○	○	○	○
The frequency of training is adequate.	○	○	○	○	○	○
Training is delivered in a format that helps me learn.	○	○	○	○	○	○
I would like to receive more euthanasia training.	○	○	○	○	○	○
Training has made me confident about performing euthanasia.	○	○	○	○	○	○
Training includes strategies to cope with personal stress.	○	○	○	○	○	○
Training includes human safety while performing euthanasia.	○	○	○	○	○	○
Training includes strategies for emotional wellness.	○	○	○	○	○	○
All employees performing euthanasia have been trained adequately.	○	○	○	○	○	○
If I have questions about euthanasia procedures, I can ask a co-worker or supervisor.	○	○	○	○	○	○
I can review euthanasia training materials on the farm whenever I need.	○	○	○	○	○	○

Start of Block: I. Questions for Management Only

Q1B How many employees are on staff currently? Please specify a number:

____

Q2B How many employees are trained to perform euthanasia? Please specify a number:

____

Q3B How many sows are at this site:

- ○ <100
- ○ 100-499
- ○ 500-999
- ○ 1000-2500
- ○ 2500+
- ○ Decline to answer
- ○ Not applicable

Q4B How often do you euthanize a **sow**?

- ☐ How many daily ____
- ☐ If not daily, how many weekly ____
- ☐ If not weekly, how many monthly ____
- ☐ If not monthly, how many annually ____
- ☐ Decline to answer
- ☐ Not applicable

Q5B How often do you euthanize a **piglet**?

- ☐ How many daily ____
- ☐ If not daily, how many weekly ____
- ☐ If not weekly, how many monthly ____
- ☐ If not monthly, how many annually ____
- ☐ Decline to answer or N/A

Q6B Do employees perform euthanasia alone or with another staff member/team? If with a staff member, identify position.

- ☐ By yourself
- ☐ With staff member ____
- ☐ Decline to answer
- ☐ Not applicable

Q7B
Who makes the decision to euthanize?

- ▢ Owner (other than yourself)
- ▢ Manager (other than yourself)
- ▢ Caretaker (other than yourself)
- ▢ You
- ▢ Veterinarian
- ▢ Other ____
- ▢ Decline to answer

Q8B What determines which euthanasia method is used?

____

____

____

____

____

Q9B
If you euthanize sows, please answer the following questions:

Questions related to type of euthanasia and frequency of use:

	Is this method used on this farm?			Frequency of Use				
	Yes	No	Decline to answer	How many daily	If not daily, how many weekly	If not weekly, how many monthly	If not monthly, how many annually	Decline to answer
Gunshot			○					
Penetrating captive bolt			○					
Electrocution			○					
Anesthetic overdose			○					

Q10B
If you euthanize sow, please answer the following questions:

Questions related to training associated with various methods of euthanasia:

	Do you provide training on	Frequency of Training	What language was the training offered in?	What languag e was	Type of training (select all that apply)	Type of training

	this method to employees?										the training offered in?									
	Yes	No	Decline to answer	How many times per year do you train employees for this type of euthanasia?	Decline to answer	Spanish	English	Both	Other	Decline to answer	If Other was selected, specify	in-person outside trainer	in-person in-house trainer	video	online	written materials	shadowing a co-worker	Other	Decline to answer	If Other was selected, specify
Gunshot																				
Penetrating captive bolt																				
Electrocution																				
Anesthetic overdose																				

Q11B

If you euthanize piglets, please answer the following questions:

Questions related to type of euthanasia and frequency of use:

	Is this method used on this farm?			Frequency of Use				
	Yes	No	Decline to answer	How many daily	If not daily, how many weekly	If not weekly, how many monthly	If not monthly, how many annually	Decline to answer
Carbon dioxide								
Non-penetrating captive bolt								
Penetrating captive bolt (>12lbs)								
Electrocution (> 3 days)								
Anesthetic overdose								
Blunt force trauma (								

Q12B

If you euthanize piglets, please answer the following questions:

Questions related to training associated with various methods of euthanasia:

	Do you provide training on this method to employees?			Frequency of Training		What language was the training offered in?					What language was the training offered in?	Type of training (select all that apply)								Type of training
	Yes	No	Decline to answer	How many times per year do you train employees for this type of euthanasia?	Decline to answer	Spanish	English	Both	Other	Decline to answer	If Other was selected, specify	in-person outside trainer	in-person in-house trainer	video	online	written materials	shadowing a co-worker	Other	Decline to answer	If Other was selected, specify

Carbon dioxide																				
Non-penetrating captive bolt																				
Penetrating captive bolt (>12lbs)																				
Electrocution (> 3 days)																				
Anesthetic overdose																				
Blunt force trauma (																				

Q13B What workplace wellness programs are in place to help promote worker health? Please describe any programs or events.

____

Q14B Is there an emphasis on mental health in the workplace?

- Yes
- No
- Decline to answer

Q15B What is the average turnover rate of employees in the past 12 months?

____

Q16B What is the most common reason for leaving? Think of the last three or four people who have left - what was their reason for leaving)

____

____

____

____

____

End of Block: I. Questions for Management Only

Q14 At your work:

				Frequency		Additional Notes Regarding Answer
	Yes	No	Decline to answer	How many take place per year?	Decline to answer	Please add any clarifying information if necessary:

Are there programs to promote worker health?			○				
Are there any mental health evaluations?			○				
Are there employee check-ins with a supervisor or administrator?			○				

Q15
If you have answered yes to any of the questions above, please explain the details of any program, evaluation or check-ins you may have at your workplace. If no, type N/A or leave blank.

____

____

____

____

Q16 Do you feel that these programs, evaluations or check-ins benefit your overall happiness in your job? Please, explain. If this does not apply to you, type N/A or leave blank.

____

____

____

____

____

Q17 The following questions relate to your employment.
Please rate your experience with each topic by checking the appropriate box.

	Strongly Disagree	Disagree	Agree	Strongly agree	Decline to answer	Not applicable
I have received guidance/advice on how to manage the stress in my workplace.	○	○	○	○	○	○
My workplace has access to programs and/or training to help me adequately deal with my work responsibilities.	○	○	○	○	○	○
I feel physically safe and protected at work while preforming euthanasia.	○	○	○	○	○	○
My job has adequate programs to help me cope with my job.	○	○	○	○	○	○
I am satisfied with my current job.	○	○	○	○	○	○
I feel supported by my peers in the workplace.	○	○	○	○	○	○

Q18
The following question relates to your employment.
Please rate your experience with each topic by checking the appropriate box.

	Not Valued	Somewhat Valued	Valued	Very Valued	Decline to answer	Not applicable
Please rate how your workplace values the mental health of employees.	○	○	○	○	○	○

Q19

The following question relates to your employment.
Please rate your experience with each topic by checking the appropriate box.

	Not Stressed	Somewhat Stressed	Stressed	Very Stressed	Decline to answer	Not applicable
Please rate the amount of stress you feel at your job on a daily basis.	○	○	○	○	○	○

Q20 Please rate your experience with each topic by checking the appropriate box.

	Strongly Disagree	Disagree	Agree	Strongly agree	Decline to answer	Not applicable
I believe that euthanasia is a humane way to end animal suffering.	○	○	○	○	○	○
It is more humane to euthanize animals that are suffering than to let them die naturally.	○	○	○	○	○	○
I feel as though the euthanasia process on the farm is necessary.	○	○	○	○	○	○
I have enough experience and knowledge to know when to euthanize a pig.	○	○	○	○	○	○
I feel as though there are often good reasons for euthanizing pigs.	○	○	○	○	○	○
I feel emotionally upset after euthanizing an animal.	○	○	○	○	○	○
Euthanizing pigs becomes easier the more that I do it.	○	○	○	○	○	○
It would not bother me if my job was to euthanize all the pigs that needed to be euthanized every day.	○	○	○	○	○	○

Q21 Is there any aspect of the euthanasia process that bothers or distresses you? Please explain.

____

____

Q22 Is there anything else you would like to share about your role in performing euthanasia?

Q23 Is there anything else you would like to share about euthanasia?

Q24 Please rate your experience with each topic by checking the appropriate box.

	Strongly Disagree	Disagree	Agree	Strongly agree	Decline to answer	Not applicable
I feel as though I can communicate with my supervisors if I feel uncomfortable performing euthanasia.	○	○	○	○	○	○
I am aware of proper channels to communicate issues to management.	○	○	○	○	○	○
My supervisors aim to promote a safe and encouraging work environment.	○	○	○	○	○	○
I feel as though my supervisors acknowledge concerns that I may have.	○	○	○	○	○	○
My supervisor takes an interest in my professional development and/or job performance.	○	○	○	○	○	○
My supervisor takes an interest in my over-all well-being.	○	○	○	○	○	○

Q25 How many people do you talk to about your work? Please specify the number of people by group.

- ○ Relatives ____
- ○ Friends ____
- ○ Work Peers ____
- ○ Other ____
- ○ Not applicable. Type an "X" ____
- ○ Decline to answer. Type an "X"

Q26 How many people do you feel you can communicate with regarding your feelings about performing euthanasia at work? Please specify the number of people by group.

- ○ Relatives ____
- ○ Friends ____
- ○ Work Peers ____
- ○ Other ____
- ○ Not applicable. Type an "X" ____
- ○ Declined to answer. Type an "X" _____________

Q27 Do you know what mental health care resources are available in your community?

- ○ Yes
- ○ No
- ○ Not applicable
- ○ Decline to answer

Q28 Please type your email if you would like to receive a gift card upon completion of the survey (optional). This email will not be used for any other purpose.

____

Q29 Age in years

____

Q30 Gender

- ○ Man
- ○ Woman
- ○ Other
- ○ Decline to answer question

Q31 Highest level of formal education

- ○ No high school diploma. Please specify number of years of schooling: ____
- ○ High school diploma
- ○ Some college
- ○ Bachelor’s degree
- ○ Veterinary school
- ○ Post graduate degree (other than veterinary school). Please specify: ____
- ○ Decline to answer question

Q32 Ethnicity

- ○ Hispanic or Latino
- ○ Non-Hispanic or Latino
- ○ Decline to answer question

Q33 How long have you been employed at this company?

- ☐ Years ____
- ☐ Month ____
- ☐ Weeks ____
- ☐ Decline to answer question

Q34 Prior to this farm, were you employed in other sow farms on the US? *If yes, total years employed in sow farms in the US:* _____ years _____months

- ○ Yes ____
- ○ No
- ○ Decline to answer question

Q35 Country of origin:

____

Q36 Native language:

____

Q37 Second language proficiency (please specify language in the textbox):

- ○ I speak it very well ____
- ○ I understand it but have trouble speaking it ___________________________________
- ○ I don’t understand it very well ____
- ○ None
- ○ Decline to answer question

Q37 Please leave any additional comments if you have any feedback or would like to discuss anything else regarding any of the questions in this survey that may not have been addressed directly.

____

____

____

____

____
